# Autosomal Recessive Hyper-IgE Syndrome in a Child With Beta Thalassemia Trait: A Case Report

**DOI:** 10.7759/cureus.61864

**Published:** 2024-06-06

**Authors:** Suresha Linganagouda, Renuka S Jadhav, Sarita Verma, Rasika S Bharaswadkar

**Affiliations:** 1 Pediatrics, Dr. D. Y. Patil Medical College, Hospital & Research Centre, Dr. D. Y. Patil Vidyapeeth (Deemed to be University), Pune, IND; 2 Pediatric Oncology, King Edward Memorial Hospital, Pune, IND

**Keywords:** genetics medical education, rare coexistence, beta thalassemia, autosomal recessive, autoimmune disorder

## Abstract

Autoimmune diseases are multifaceted disorders, and their coexistence with other conditions can present unique challenges in diagnosis and management. Here, we report a rare case of autosomal recessive hyper-IgE syndrome (AR-HIES) in a child with beta thalassemia trait. AR-HIES is a distinct immunodeficiency disorder characterized by severe eczema and recurrent bacterial and viral infections, particularly affecting the sinopulmonary system. This case highlights the importance of recognizing and managing the co-occurrence of rare genetic conditions, as it can impact treatment strategies and familial counseling. This unique case of AR-HIES in a child with beta thalassemia trait underscores the complexity of autoimmune disorders and the need for comprehensive evaluation in patients presenting with multiple clinical manifestations.

## Introduction

It is estimated that more than six million people are affected with a primary immunodeficiency disorder, out of which 70% to 90% remain undiagnosed [1]. The hyper-IgE syndromes (HIES) are rare kinds of primary immunodeficiencies that were first described by Davis, Schaller, and Wedgwood in 1966 [2]. Although sporadic cases of HIES are prevalent, both autosomal dominant (AD) and recessive inheritance patterns are not uncommon. Patients frequently experience recurring infections, pneumonia, and elevated blood IgE levels [3]. AD-HIES, also known as Job syndrome, is caused by a mutation in the signal transducer and activator of transcription-3 (STAT3). This syndrome is characterized by a heightened susceptibility to recurring bacterial and fungal infections as well as an increased likelihood of developing pneumonia and pneumatocoeles [4,5].

Primary immunodeficiency disorders are increasingly being recognized in children owing to the accessibility and availability of genetic tests. These disorders form a large group of previously undiagnosed entities and warrant early suspicion and intervention for definitive treatment. Multiple criteria have been put forward for guiding the approach to diagnosis and treatment of a suspected case of primary immunodeficiency [1]. The spectrum ranges from mild defects to life-threatening conditions like severe combined immunodeficiency (SCID). Accordingly, the treatment also ranges from lifestyle modification with appropriate prophylaxis to bone marrow transplant (BMT) [6]. Understanding the diverse clinical manifestations associated with these disorders facilitates their timely diagnosis and management. We describe our clinical experience of diagnosis and treatment of a case of autosomal recessive HIES (AR-HEIS) in a child which is characterized by increased vulnerability to viral infections and severe atopic eczema.

## Case presentation

A five-year-old female child born out of a third-degree consanguineous marriage, presented with complaints of exanthematous rash, associated with itching and bloody discharge from the lesions, first noticed over the abdomen and later started appearing all over the body. Since 18 months of age, the child had such episodes on and off, relieved temporarily for a short duration of one to two weeks by local medications, following which it would recur.

**Figure 1:**
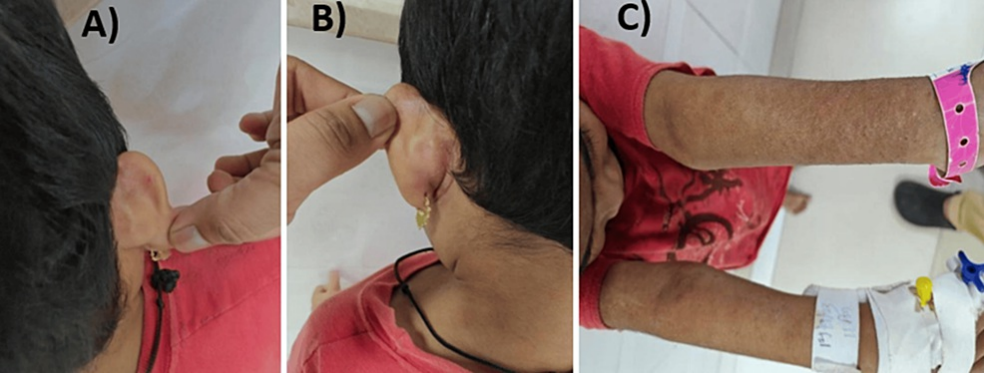
Clinical images of the patient pretreatment B/L post auricular rash (A, B). Background atopy with erythematous rash (C)

The child was admitted multiple times (3-4 times in a year) with lower respiratory tract infection (LRTI) and recurring episodes of otitis media since infancy. She had severe anemia at the age of 18 months and was diagnosed with beta thalassemia trait at 21 months of age (via hemoglobin (Hb) electrophoresis). However, her other complaints continued to persist and recur. She presented currently with mainly dermatological complaints (Figure 1) and respiratory tract infection. She was immunized till her present age and was developmentally normal. There was no significant positive family history. On general examination, pallor and bilateral cervical lymphadenopathy were present. Bacillus Calmette-Guérin (BCG) scar was present. Anthropometric measurements of the child were normal with normal dentition. Her systemic examination was within normal limits.

**Figure 2:**
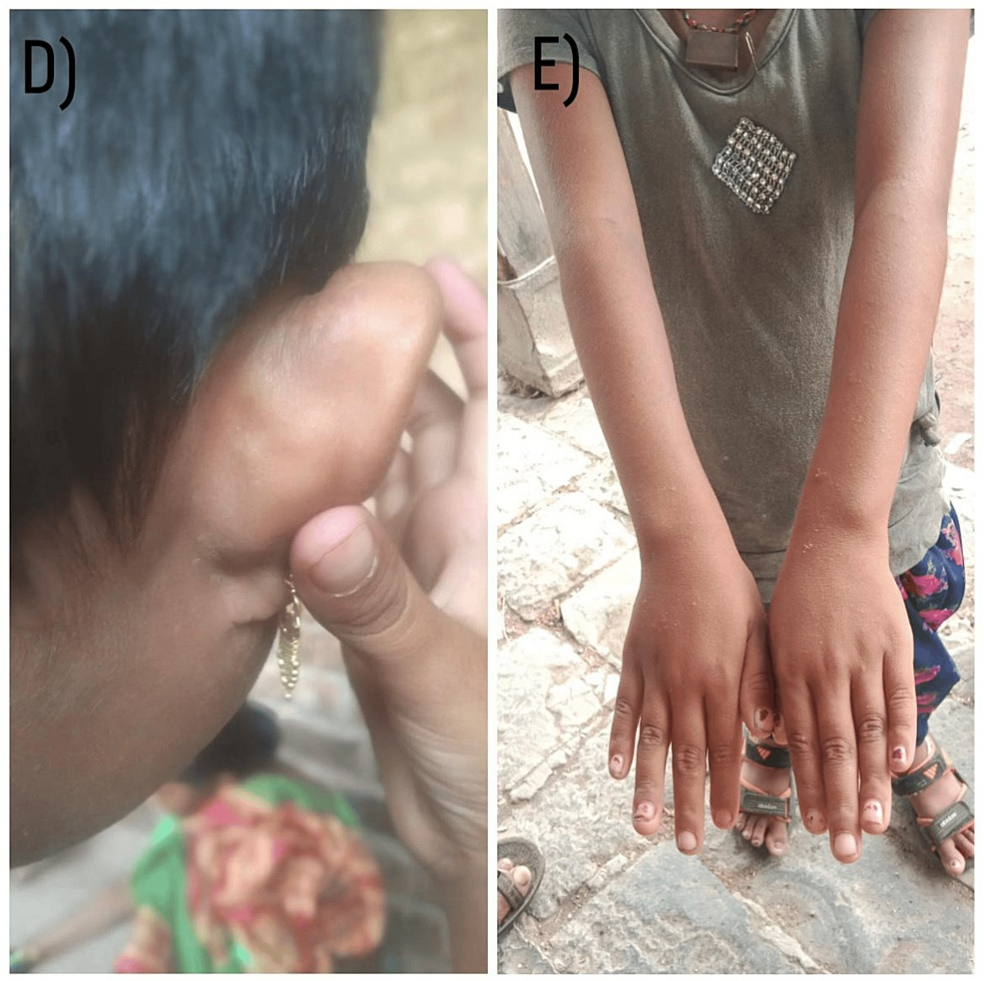
Clinical images of the patient at six-month follow-up Resolved atopy and rash (D, E)

The child’s initial investigations revealed eosinophilic leukocytosis with an absolute eosinophilic count (AEC) of 15,792. In view of rash, recurrent otitis, and eosinophilia with recurrent LRTI, the clinical differential diagnosis was Langerhans cell histiocytosis and primary immunodeficiency disorder. Skin biopsy showed no classical Langerhans cells and dermo-epidermal junction inflammation or eosinophilic infiltrates were also not present. Parents did not consent to the bone marrow procedure. Serum immunoglobulin levels revealed markedly elevated IgE levels (Table 1). The family agreed to whole exome sequencing that revealed heterozygous likely pathogenic variant and variant of uncertain significance (VUS) in DOCK-8 gene consistent with phenotype detected (primary immunodeficiency phenotype; hyper-IgE recurrent infection syndrome 2, autosomal recessive) along with heterozygous pathogenic variant in HBB gene, consistent with the detected phenotype (beta thalassemia).

**Table 1:** Laboratory investigations

Investigation	Result	Reference range
Hemoglobin	8.70	11.8-14.7 g/dl
White blood cell (WBC)	32,900	4,000-12,000 /uL
Platelet count	460,000	15,000-410,000 /uL
Absolute neutrophil count (ANC)	10,199	1,500-8,500 /uL
Absolute leukocyte count (ALC)	5,593	1,500-7,000 /uL
Absolute eosinophil count (AEC)	15,792	0-650 /uL

The patient underwent treatment with intravenous antibiotics, alongside oral and topical corticosteroids for the management of atopic eczema, resulting in substantial alleviation of symptoms. Tablet prednisolone was administered at a dosage of 1 mg/kg/day for a duration of two weeks, followed by a gradual tapering over an additional two-week period. Furthermore, tablet hydroxyurea was initiated, resulting in normalization of absolute eosinophil count (AEC) after four weeks and continued for a total duration of six months, as there are no standard guidelines regarding treatment duration. Lifestyle modifications were implemented, and scheduled regular follow-ups were conducted. Initially, the patient followed-up every four weeks for two months, subsequently transitioning to a follow-up frequency of every three months. Notably, throughout the past six months, the patient remained free from episodes necessitating hospitalization.

## Discussion

There have been major advances in the understanding, diagnosis, and treatment of the vast spectrum of primary immunodeficiency disorders. With the advent of genetic tests, more than 400 types of these disorders have been documented. The diagnosis has seen an upsurge in the last decade enabling more accurate and timely identification of patients with immunodeficiencies [7]. However, despite these advancements, several challenges remain in achieving a definitive diagnosis.

AR-HIES is characterized by a heightened vulnerability to viral skin infections and more severe cases of atopic eczema [8]. The presence of skin rash with a history of recurrent infections prompted us to consider a differential diagnosis of primary immunodeficiency. The primary cause of this disease is genetic mutations in the tyrosine kinase 2 gene (TYK2) and dedicator of cytokinesis 8 (DOCK8) gene, with the DOCK8 mutation being more common. DOCK8 deficiency manifests with a variety of symptoms, including the development of severe viral infections of the skin like warts and molluscum, as well as a heightened susceptibility to cancer at a younger age. People with TYK2 deficiency exhibit a unique manifestation of BCG infection [9]. None of these manifestations were found in our case; however, the child will be followed up closely as these symptoms can evolve over time.

Although the cause of most AR-HIES cases is still unknown, it is believed to be due to autosomal recessive inheritance, as indicated by consanguinity and the presence of multiple affected siblings. The laboratory findings that are most reliable and easily observed are elevated levels of eosinophils and serum IgE, which may be more pronounced when compared to AD-HIES [8]. Our patient also had these classical laboratory findings of hypereosinophilia and elevated IgE levels.

Though autoimmune cytopenias can manifest as symptoms, previous studies in immunology have shown that lymphocyte phenotyping does not yield different outcomes. AR-HIES is a unique medical condition characterized by increased levels of IgE, recurring skin and lung infections, eczema, vulnerability to viral infections such as molluscum contagiosum, and central nervous system involvement that lacks a precise definition. However, it does not exhibit the typical skeletal, dental, and connective tissue defects observed in AD-HIES [10]. Our patient needs to be followed up for manifestations of these conditions in the future.

Curative treatment of AR-HES is possible only with hematopoietic stem cell transplantation (HSCT) [11]. However, no standard recommendation exists to select the patients for BMT. Pharmacological treatment of eczema and lung conditions with dupilumab has shown promising results in certain cases [11]. As our patient could not afford both these options we managed the child with topical emollients and oral steroids empirically. The eczema responded after two weeks and the eosinophilia improved with resolution of the respiratory complaints. The child is presently on Septran and antifungal prophylaxis with topical emollients and lifestyle modifications for the last six months, and there have been no breakthrough infections.

The thalassemia trait was an incidental occurrence in our patient; however, this highlights the possibility of multiple genetic mutations in the same child. Various other genetic disorders have been reported concurrently with beta thalassemia trait [12]; however, in this case report, AR-HIES was found to be present along with beta thalassemia trait. The presence of multiple genetic mutations underscores the importance of genetic counseling, which becomes both crucial and challenging for the family. In our case, the family knew about the thalassemia trait status of the child since at a young age and now were disclosed about another genetic problem. Long-term compliance becomes challenging in such scenarios though they are explained about the mildness of one mutation and implications of the second mutation. Our patient also needs repeated reinforcement for regular follow-up.

## Conclusions

AR-HIES is a rare type of primary immunodeficiency syndrome. Early suspicion and prompt diagnosis are crucial for preventing severe life-threatening infections leading to better outcomes and counseling about long-term prognosis and follow-up. These can become chronic, generate long-lasting sequelae, and prove to be life-threatening if not managed adequately. Inborn errors of immunity are known for their association with malignancies and other immunological issues such as allergies, autoimmunity, and inflammation. Addressing only the symptoms without investigating the underlying cause can exacerbate the patient's overall health. Recognizing the expanding spectrum of manifestations associated with primary immunodeficiency disorders is essential in mitigating the long-term consequences of the condition.
